# Polyphyllin VI screened from Chonglou by cell membrane immobilized chromatography relieves inflammatory pain by inhibiting inflammation and normalizing the expression of P2X_3_ purinoceptor

**DOI:** 10.3389/fphar.2023.1117762

**Published:** 2023-02-14

**Authors:** Zhenhui Luo, Tingting Wang, Zhenglang Zhang, Hekun Zeng, Mengqin Yi, Peiyang Li, Jiaqin Pan, Chunyan Zhu, Na Lin, Shangdong Liang, Alexei Verkhratsky, Hong Nie

**Affiliations:** ^1^ International Cooperative Laboratory of Traditional Chinese Medicine Modernization and Innovative Drug Development of Chinese Ministry of Education (MOE), College of Pharmacy, Jinan University, Guangzhou, China; ^2^ Guangdong Province Key Laboratory of Pharmacodynamic Constituents of TCM and New Drugs Research, College of Pharmacy, Jinan University, Guangzhou, China; ^3^ Institute of Chinese Materia Medica, China Academy of Chinese Medical Sciences, Beijing, China; ^4^ Neuropharmacology Laboratory of Physiology Department, Basic Medical School, Nanchang University, Nanchang, Jiangxi, China; ^5^ Faculty of Biology, Medicine, and Health, The University of Manchester, Manchester, United Kingdom

**Keywords:** Chonglou, polyphyllin VI, chronic neuroinflammatory pain, P2X3 receptor, cell membrane immobilized chromatography

## Abstract

**Objective:** Inflammatory pain is one of the most common diseases in daily life and clinic. In this work, we analysed bioactive components of the traditional Chinese medicine Chonglou and studied mechanisms of their analgesic effects.

**Material and methods:** Molecular docking technology and U373 cells overexpressing P2X3 receptors combined with the cell membrane immobilized chromatography were used to screen possible CL bioactive molecules interacting with the P2X3 receptor. Moreover, we investigated the analgesic and anti-inflammatory effects of Polyphyllin VI (PPIV), in mice with chronic neuroinflammatory pain induced by CFA (complete Freund’s adjuvant).

**Results:** The results of cell membrane immobilized chromatography and molecular docking showed that PPVI was one of the effective compounds of Chonglou. In mice with CFA-induced chronic neuroinflammatory pain, PPVI decreased the thermal paw withdrawal latency and mechanical paw withdrawal threshold and diminished foot edema. Additionally, in mice with CFA-induced chronic neuroinflammatory pain, PPIV reduced the expression of the pro-inflammatory factors IL-1, IL-6, TNF-α, and downregulated the expression of P2X3 receptors in the dorsal root ganglion and spinal cord.

**Conclusion:** Our work identifies PPVI as a potential analgesic component in the Chonglou extract. We demonstrated that PPVI reduces pain by inhibiting inflammation and normalizing P2X3 receptor expression in the dorsal root ganglion and spinal cord.

## 1 Introduction

Pain, and the inflammatory pain in particular, is common in daily life and clinical practice, (Taneja et al., 2017). (Ronchetti et al., 2017). Even after resolution of inflammation, chronic pain can develop and last for a long period (Demir et al., 2013; Raja et al., 2020; Finnerup et al., 2021). Over 7% of the world’s population is estimated to have chronic pain with neuropathic symptoms, however, due to the challenges in categorization and the incomplete understanding of the underlying processes, this proportion may be understated (van Hecke et al., 2014; Bouhassira, 2019).

Neuroinflammation that can affect the peripheral or central nervous system is primarily defined by the infiltration of leukocytes, reactive gliosis, and the upregulation of inflammatory mediators (Escartin et al., 2021). It is generally believed that inflammation is crucial in the maintenance and management of chronic pain (Vergne-Salle and Bertin, 2021). Findings from fundamental studies utilizing chronic pain-prone animals demonstrated that chronic pain results from a pathologically altered neural circuits evoked by peripheral tissue inflammation and peripheral nerve damage (Malcangio, 2019). ATP promotes nociceptive processing by activating the ligand-gated ion channel family of P2X receptors, among which, the P2X_3_ receptor, is highly expressed by primary afferent neurons. In sensory neurons, P2X_3_ receptors function as homomeric (P2X_3_) and heteromeric (P2X_2/3_) channels (Jarvis, 2003). Exogenous application of ATP and related agonists excites the peripheral and central nervous system and increases sensitivity to noxious stimuli. Specific targeting of the P2X_3_ receptor by genetic deletion and knockdown results in a hypoalgesic phenotype (Butler and Meegan, 2008). Studies have shown that the pharmacological blockade of P2X_3_ receptors completely blocked specific types of chronic inflammatory and neuropathic pain (Kaan et al., 2010; Xu et al., 2012; Jorge et al., 2020). Peripheral nerve injury differentially alters the functional expression of P2X_3_ receptors in small- and large-diameter primary afferent neurons (Inoue, 2021). As a result, P2X_2,3_ purinoceptors can represent targets for pain therapy.

*Chonglou* (CL) is the dried rhizome of *Paris polyphylla* var. *yunnanensis* or *P. polyphylla ar. chinensis*. Steroidal saponins, flavonoids, sugars, volatile oils, amino acids, trace minerals, etc., are among CL’s active constituents, mediating anti-tumor (Tian et al., 2020), anti-infection (Qiumin et al., 2017), organ-protecting (Man et al., 2014), and anti-inflammatory effects (Yan et al., 2021; Zhou et al., 2021). Several active compounds of CL such as polyphyllin D, polyphyllin A, polyphyllin I, polyphyllin II, polyphyllin VI (PPVI), and polyphyllin VII were identified (Wang Q. et al., 2018; Pang et al., 2020; Teng et al., 2020; Ahmad et al., 2021; Kwon et al., 2021). Bioactive chemicals from CL for the treatment of inflammatory pain and the underlying mechanisms were not fully elucidated.

**FIGURE 1:**
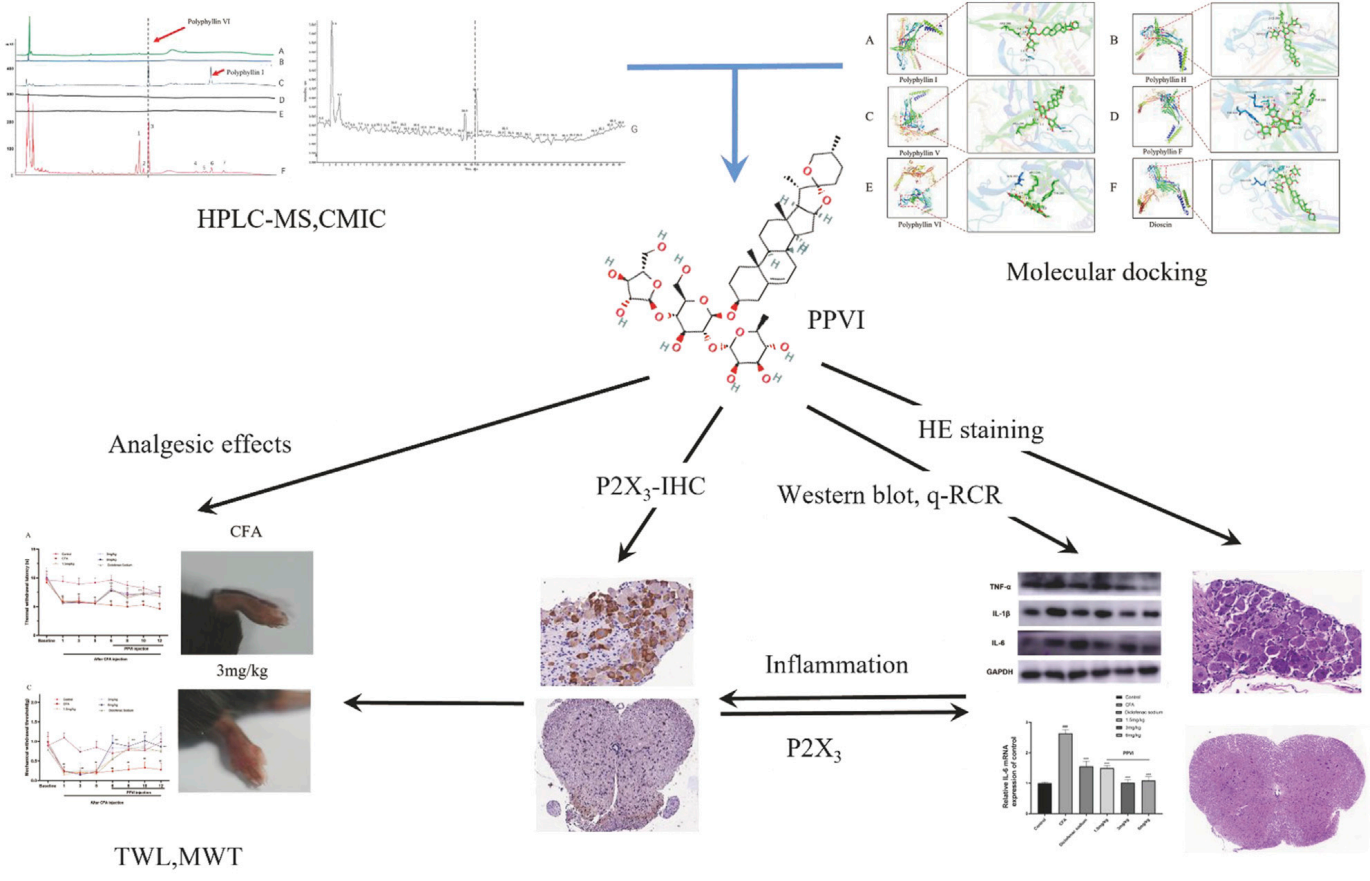
The flowchart the article. Polyphyllin VI was screened from CL by CMIC and relieved the chronic inflammatory pain *via* suppressing P2X_3_R in the following experiments.

The development and use of traditional Chinese medicines (TCM) are significantly hampered by the difficulty in identifying active compounds among the hundreds of ingredients in medicinal formulae. A novel screening technique named Cell Membrane Immobilization Chromatography (CMIC), based on the biospecific affinity adsorption of biologically active substances to receptors or channels in cells was developed for isolating active compounds from the natural samples (Nie et al., 2008; Nie et al., 2011; Zhang et al., 2021). In this study, we used cell membrane immobilized chromatography (CMIC) to screen the active ingredients in CL that may interact with the P2X_3_ receptor. We identified PPVI as the main active ingredient of the CL extract and investigated the analgesic potency and mechanism of PPVI by molecular docking combined with CMIC on U373 cells expressing P2X receptors. The flowchart in Figure 1 overviews technical procedures and experimental outcomes for assessing PPVI’s impact and mechanisms of action.

## 2 Material and methods

### 2.1 Preparation and extraction of CL

CL was purchased from the First Affiliated Hospital of Jinan University and certified by Professor Nie Hong. The drugs were pulverized to a fine powder (30 mesh) by the grinder, 1.25 g of fine powder was dissolved in 50 mL of analytical methanol (99.9%), and sonicated for half an hour under ultrasonic conditions (60°C, 39.6 kHz) to obtain 25 mg/mL CL decoction liquid. The medicinal liquid of the decoction was filtered by double-circle quantitative filter paper and passed through a 0.22 μm microporous membrane to obtain the CL decoction liquid sample. Polyphyllin I (B21668, Polyphyllin II (B21669 and Polyphyllin VI (B21670) were purchased from Shanghaiyuanye Bio-Technology Co. Ltd.; the purity of each reference compound was greater than 98%, which was evaluated by analytical high-performance liquid chromatography combined with diode array detection and mass spectrometry (HPLC-DAD-MS).

### 2.2 HPLC with mass spectrometry

HPLC-DAD analysis using Agilent 1200 series was equipped with ChemStation software (Agilent Technologies, Valderbrunn, Germany). Chromatographic separation was performed on a ChromCoreTM 120 C18 column (laboratory technology NanoChrom, Jiangsu, China) with a diameter of 4.6 × 250 mm and a length of 5 μm. The mobile phase consisted of 0.1% aqueous ammonia (A) and acetonitrile (B). The following gradient elution procedure is used for separation: 0–40 min, 30%–60% B; 40–50 min, 60%–30% B; then balance for 10 min. The flow rate was 1 mL/min and the column temperature was maintained at 30°C. The DAD is set to scan from 190 to 400 nm. The separated compound was detected at 203 nm. Agilent 3500 TOF/MS (Agilent Technologies, Santa Clara, California, United States) equipped with electrospray ionization (ESI) interface for HPLC-DAD-TOF/MS analysis. ESI mass source spectrometers operate in negative and positive ion modes. Operating parameters are set as follows: dry gas temperature, 325°C; dry gas (N2) flow rate, 11.0 L/min; atomizer, 30 PSIG; Fragmentation voltage, 175 V; and capillary voltage, 3500 V. The range is set to 100–1000 m/z. Data acquisition and analysis were performed using Masshunter Workstation software (version B.02.00, Agilent Technologies, Inc., Waldbronn, Germany).

### 2.3 Cell membrane immobilized chromatography

U373 cells (College of Pharmacy, Jinan University) were cultured in Dulbecco’s modified Eagle’s media with 10% fetal bovine serum (v/v), 1% 100 U/mL penicillin, and 100 μg/mL streptomycin to perform CMIC. A 37°C humidified incubator with 5% carbon dioxide (CO_2_) was used to keep U373 cells. U373 cells were plated in a cell culture flask (25 cm^2^) cultivated until confluency was achieved. For 1 h, U373 cells were cultured in a humidified incubator with 5% CO_2_ at 37°C and 2 mL of CL water extracts (5 g/L). To get rid of any potential non-selectively combining elements, the CL water extracts in the flask were removed. The flask was then rinsed five times with 1 mL of phosphate-buffered saline (PBS). Washing duration was tuned by analyzing CL in a separate washing eluate because full removal of components that are not particularly binding is essential. For analysis using HPLC-DAD-TOF/MS (HPLC-DAD-coupled with diode array detection and time of flight mass spectrometry), the eluate from the fifth washing was collected. The last phase involved denatured U373 cells and the dissociation of associated chemicals by co-incubation with 2 mL of methanol for 30 min. Using a Termovap sample concentrator, the attached ingredients solution was evaporated to 500 μL at room temperature. The material that had evaporated was analyzed using HPLC-DAD-TOF/MS. The CMIC was described in detail previously (Zhang et al., 2021).

### 2.4 Molecular docking simulation

Using Open Babel 2.4.1, the molecular structure file of PPVI was downloaded and converted to PDBQT format after being obtained and downloaded from PubChem (https://pubchem.ncbi.nlm.nih.gov/). The crystal structure of the target protein was then retrieved and downloaded from the RCSB Protein Data Bank database (RCSBPDB, https://www.rcsb.org/). Using AutoDock Tools (Trott and Olson, 2010), the target protein’s ligands and water were eliminated, yielding a new protein. It simultaneously determines the size and center of the docking box, calculates charge, inserts hydrogen atoms, outputs the PDBQT format file, and all of these things. Vina was used to dock the active ingredients with the target protein one at a time, choosing the conformation with the best docking score (Affinity). The outcomes were then examined using Pymol, which produced graphs.

### 2.5 Animals

6–9 weeks adult female C57BL/6 mice (20–25 g) were obtained from Guangdong Yaokang Biotechnology Co., Ltd. They were housed in Jinan University Laboratory Animal Center at a standard temperature of 24°C ± 1°C under a 12 h light-dark cycle (dark from 7:00 p.m. to 7:00 a.m.) with free access to food and water. All experiments were conducted by the National Institutes of Health Guide for the Care and Use of Laboratory animals in rigorous line with the International Association for the Study of Pain guidelines. Our research was approved by the Jinan University Animal Ethics Committee. The behavioral test was performed by experimenters who were blinded to the experimental group.

### 2.6 Construction of CFA model and drugs administration

The mechanical withdrawal threshold (MWT) and the thermal paw withdrawal latency (TWL) of C57BL/6 mice were measured after the mice were placed in the animal room for a week to adjust to the environment. Mice were then placed on a metal mesh, covered with plexiglass, and tested. Mice with no significant differences were selected according to MWT and TWL, and divided into six groups (*N* = 10) using a random number table. The following experimental groups were set: Control group, CFA model group, and CFA model + different dosages of Polyphyllin by intraperitoneal injection (1.5, 3, and 6 mg/kg, respectively designated as PPVI-1.5, PPVI-3, and PPVI-6 groups); The CFA modeling approach including three steps were used. The mouse left hind foot was injected with 30 μL of CFA, the mice pain threshold was measured in Von-Frey nylon silk and hot plate protocols, and the mice ability to tolerate pain was assessed. Based on the mice ability to tolerate pain, the validity of the CFA model was judged. The diclofenac sodium (7.5 mg/kg) was administered by intraperitoneal injection following CFA modeling. Five days after the CFA model was established drugs were administered once per day for a total of 7 days. Carbon dioxide was utilized for euthanasia after the experiment.

### 2.7 The von Frey test

After 30 min of acclimatization, mice were tested using von Frey hair (0.04 g, 0.07 g, 0.16 g, 0.40 g, 0.60 g, 1.00 g, 1.40 g, and 2.00 g), which was slightly bent to stimulate the lateral part of the mouse’s left plantar. The mice were placed on an elevated mesh metal plate, covered with perforated transparent 10 cm × 6 cm × 6 cm plexiglass cages. Special emphasis was taken to separate pain-induced withdrawal behavior from the withdrawal response following physical exercise. If the stimulus is positive, it is recorded with an X; if it is negative, it is marked with an O. After changing the O to X or X to O, the above procedure is repeated for four rounds, with the pressure value indicated by the letter X (the last used fiber) being recorded after each round. If there is no response, the level of pressure is increased to the next level, the first mechanical stimulation is given with a force of 1.0 g, and so on. The formula for calculating the threshold value is as follows: Log 50%g threshold = X_f_ + kδ (X_f_ = value (in log units) of the final von Frey hair used; k = tabular valufor the pattern of positive/negative responses; and *δ* = mean difference (in log units) between stimuli (Chaplan et al., 1994).

### 2.8 The hot-plate test

Before the experiment, mice were prescreened by having their abdominal hair removed, and being placed one at a time on a (55.00.5) °C hot plate apparatus. The pain thresholds were recorded using hind feet licked or lifted as pain indicators. Mice with a pain threshold of 5–30 s were chosen for the experiment. The selected mice were placed one at a time on the hot plate device, and the mice pain threshold was measured three times, with 10-min intervals between each measurement. The average value was taken as the baseline pain threshold or the pre-administration, normal pain tolerance. The average time that mice spend licking their paws on a hot plate after taking PPVI, which correlates to the mice’s response to heat stimulation after taking PPVI, is measured as the pain threshold. The pain thresholds before and after administration were determined and counted for the 60 s if the mice on the hot plate equipment still did not exhibit any signs of discomfort after the 60 s.

### 2.9 The measurement of foot swelling

Vernier calipers were used to measure each group of mice toe thickness before modeling, during modeling, and after drug treatment. The difference between the thickness of the left and right hind limbs was utilized to indicate the degree of inflammation and swelling. Each group’s degree of swelling was calculated.

### 2.10 RT-qPCR

Using Trizol reagent (Thermo Fisher), total RNA was obtained from L_3_-L_5_ DRG and the spinal cord. The concentration and purity of extracted RNA were assessed using a spectrophotometer. For qPCR, RNA with an approximate absorbance ratio of 2.0 (OD260/OD280 nm) was selected, and RT Master Mix for qPCR II was used to transcribe the RNA into cDNA. Quantitative real-time PCR (RT-qPCR) was performed to measure the expression of the mRNA using a qPCR PreMix (SYBR Green) Kit. The test’s primers are listed in Table 1. The relative expressions of the relevant genes were calculated using the 2^−ΔΔCT^ method.

**TABLE 1:** Primer sequences for RT-qPCR.

Name	Sequence (5′−3′)
IL-1β (Forward sequence)	GCA​ACT​GTT​CCT​GAA​CTC​AAC​T
IL-1β (Reverse sequence)	ATC​TTT​TGG​GGT​CCG​TCA​ACT
IL-6 (Forward sequence)	TAG​TCC​TTC​CTA​CCC​CAA​TTT​CC
IL-6 (Reverse sequence)	TTG​GTC​CTT​AGC​CAC​TCC​TTC
TNF-α (Forward sequence)	CCC​TCA​CAC​TCA​GAT​CAT​CTT​CT
TNF-α (Reverse sequence)	GCT​ACG​ACG​TGG​GCT​ACA​G
P2X_3_ (Forward sequence)	AAA​GCT​GGA​CCA​TTG​GGA​TCA
P2X_3_ (Reverse sequence)	CGT​GTC​CCG​CAC​TTG​GTA​G
*β*-Actin (Forward sequence)	GTG​ACG​TTG​ACA​TCC​GTA​AAG​A
*β*-Actin (Reverse sequence)	GCC​GGA​CTC​ATC​GTA​CTC​C

### 2.11 Western blot

Protein extraction was described by (Luo et al., 2021). Sodium dodecyl sulfate-polyacrylamide gel electrophoresis (SDS-PAGE) was used to separate 20 µg of proteins from each sample, which was then placed onto a polyvinylidene fluoride (PVDF) membrane. Before the primary antibody incubation at 4°C overnight, the membranes were blocked with 5% milk for 2 h. The following primary antibodies were then used: P2X_3_ (Abcam, ab300493), IL-1 (Abcam, ab283818), GAPDH (Abcam, ab9485), TNF-α (Abcam, ab215188), and IL-6 (Abcam, ab290735). Horseradish peroxidase (HRP)-conjugated secondary antibody was applied to the blotted PVDF membrane following the primary antibody reaction (Boster Biological Technology Co. Ltd.). The ECL chemiluminescence western blot detection technique was carried out *via* the use of a gel imaging device and ImageJ.

### 2.12 Histology and immunohistochemistry

#### 2.12.1 Hematoxylin-eosin staining

The DRG and spinal cord tissue of the L_3_-L_5_ segment of mice were fixed in 4% paraformaldehyde for 24 h, dehydrated in the order of 75%, 85%, and 95% alcohol for 2 h, dehydrated with absolute ethanol I, II for 30 min. Anhydrous ethanol-xylene and xylene were dehydrated for 10 min respectively, and then transferred to 58°C paraffin wax I, II, and III for 1 h. Tissues were embedded using a Leica embedding machine and then placed in a −20°C freezer until the paraffin solidified. The DRG and spinal cord were cut into 5 μm thick wax slices with a microtome, placed in a 60 oven for 20 min, and then soaked in xylene for 20 min to completely remove the remaining paraffin; The DRG and spinal cord slices were restored the tissue water in absolute ethanol, 95%, 85%, 75%, 50%, rinsed for 3 min and put in hematoxylin staining for 3 min, rinsed with tap water for 3 min, exposed to 1% alcohol hydrochloric acid to differentiate for 5 s, and waited for the tissue to turn red before rinsing in distilled water. Hematoxylin and eosin-stained sections of the DRG and spinal cord were observed under an optical microscope and scanned with a pathological slide scanner.

#### 2.12.2 Immunohistochemistry

Mice underwent intra-cardiac perfusion with 4% paraformaldehyde for tissue fixation. L_3_-L_5_ segment DRGs and spinal cord were dissected and placed in 4% paraformaldehyde, fixed at room temperature for 6 h. Fixed DRG and spinal cord preparations were cut into 5um thick slices and dried in an incubator set to 50°C to 60°C for 20 min. The slices were subjected to a 5-min soak in absolute ethanol twice, followed by 2-min soaks in 95%, 80%, and 70% ethanol, and 5 min in distilled water. 3% H_2_O_2_ solution was added for 10 min. Subsequently, DRG and spinal cord slices were washed three times and rinsed with PBS for 5 min. Goat serum was added for blocking for 45 min. Slices were incubated with primary antibody against P2X_3_ receptors (Abcam, ab300493) at 1: 200 dilutions overnight at 4°C, washed, incubated with HRP-conjugated secondary antibody (goat polyclonal; Abcam; 1: 200) for 40 min, washed, soaked in DAB solution for 3 min, mounted on neutral gum and observed under an optical microscope and scanned with a pathological slide scanner.

### 2.13 Statistical analysis

The means and standard error of means (SEM) for each outcome are shown. The GraphPad Prism 8.0 program was used for statistical analysis. Independent-sample t-tests were used to assess potential differences between any given pair of groups. Tukey’s *post hoc* analysis was performed to examine differences between any two groups using a one-way analysis of variance (ANOVA). Two-way repeated ANOVA was used to compare different groups, and then Tukey’s *post hoc* analysis was conducted. The *p* < 0.05 significance level was used for the whole experiment.

## 3 Results

### 3.1 Screening and identification of active compounds in PPVI using cell membrane immobilized chromatography

**FIGURE 2:**
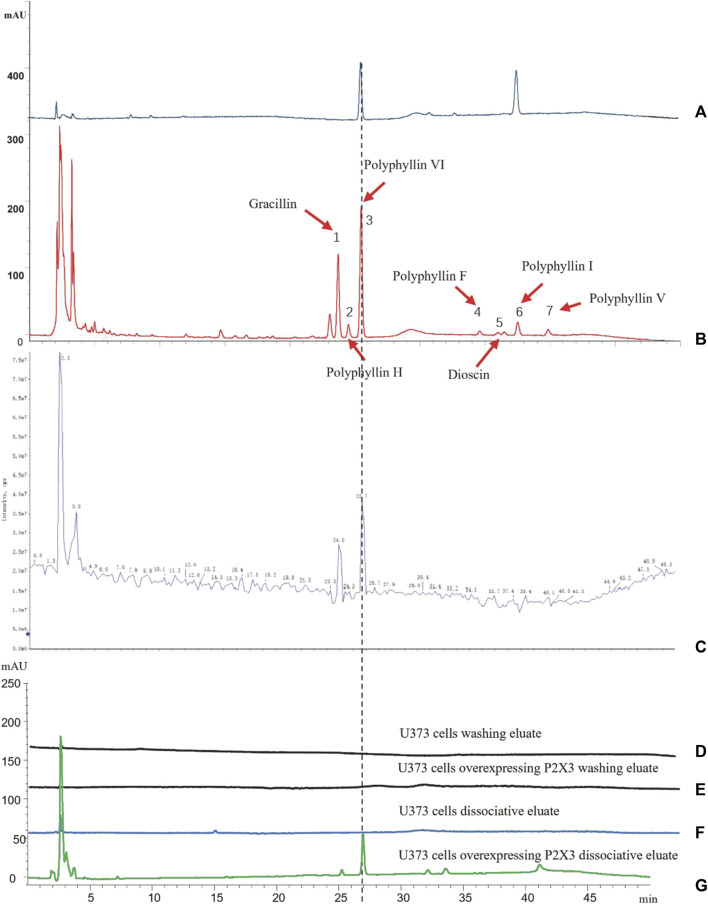
The CMIC of CL with U373 cells. **(A)** The HPLC chromatogram of the standard solution (Polyphyllin VI, Polyphyllin I). **(B,C)** THe HPLC-MS chromatogram of CL extract. **(D,E)** The HPLC chromatogram of the eluate of U373 cells and U373 cells expressing P2X_3_. **(F,G)** The HPLC chromatogram of dissociation medium of U373 cells and U373 cells expressing P2X_3_.

First, we identified the primary components of the CL extract by HPLC-MS. Seven primary peaks were discovered in the CL extract HPLC with two peaks matching the standard solution PPVI and PPI (Figures 2A, B). CL was further analyzed with HPLC-DAD-TOF/MS to reveal these substances in the CL extract (Figure 2C), identified by comparing the retention time (tR), UV absorption traits, and mass spectra with those in the literature and/or those of known reference compounds (Qin et al., 2018; Guan et al., 2021; Xie et al., 2021) to provide a preliminary compounds description (Table 2). These ingredients are Gracillin, Dioscin, Polyphyllin H, Polyphyllin I, Polyphyllin V, Polyphyllin VI, and Polyphyllin F. Second, we compared the components of U272 cells before and after overexpressing the P2X_3_ receptor, and screened out the components with the greatest difference. No peaks were seen in the eluate of the sixth washing (Figures 2D, E). However, in the dissociative eluate of the U373 cells overexpressing P2X_3_, the PPVI response value in the HPLC was relatively increased compared with the control U373 cells (Figures 2F, G). These results indicated that PPVI could interact with P2X_3_ receptors.

**TABLE 2:** HPLC-MS chromatogram of CL extract compounds identification.

NO.	Expected RT	Retention time	Retention time Delta (min)	Adduct/Charge	Formula	Precursor mass	Found at mass	Mass error (ppm)	Component name
1	24.9	24.9	0	[M-H]^-^	C_45_H_72_O_17_	883.47	883.4703	0.7	Gracillin
2	25.74	25.74	0	[M-H]^-^	C_44_H_70_O_17_	869.454	869.4521	−2.2	Polyphyllin H
3	26.65	26.65	0	[M-H]^-^	C_39_H_62_O_13_	737.412	737.4112	−0.8	Polyphyllin VI
4	35.61	35.69	0.08	[M-H]^-^	C_51_H_82_O_20_	1013.533	1013.5243	−8.3	Polyphyllin F
5	37.07	37.1	0.03	[M-H]^-^	C_45_H_72_O_16_	867.475	867.4784	4.2	Dioscin
6	38.56	38.6	0.04	[M-H]^-^	C_44_H_70_O_16_	853.459	853.4587	−0.4	Polyphyllin I
7	40.96	40.95	0.01	[M-H]^-^	C_39_H_62_O_12_	721.417	721.4157	−1.6	Polyphyllin V

### 3.2 Molecular docking of PPVI and P2X_3_ receptor

**FIGURE 3:**
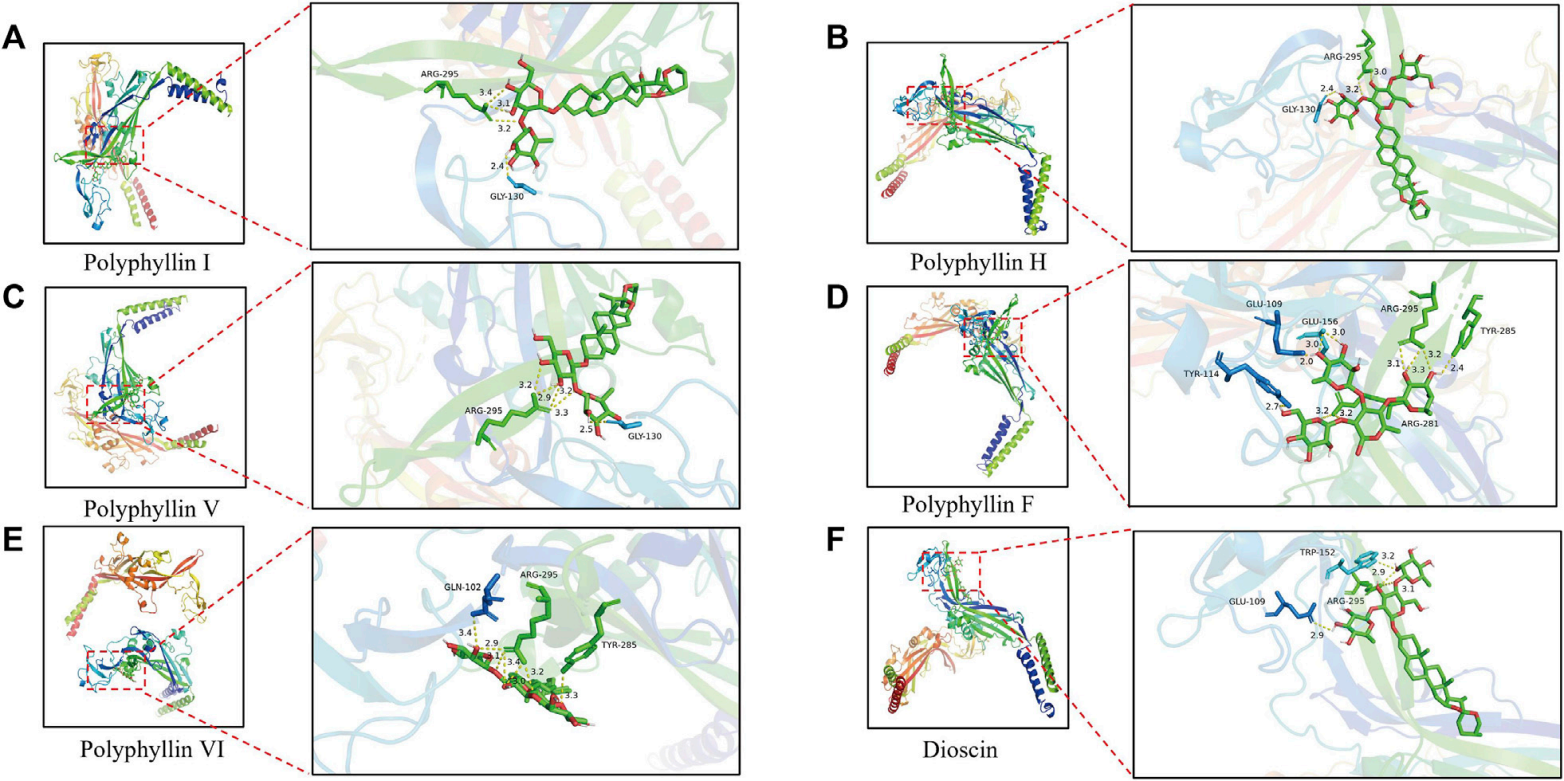
Molecular docking models of P2X_3_ and Dioscin, Polyphyllin H, Polyphyllin I, Polyphyllin V, Polyphyllin VI, Polyphyllin F. **(A)** P2X_3_ and Polyphyllin I docking mode and the interaction plane diagram. **(B)** P2X_3_ and Polyphyllin H docking mode and the interaction plane diagram. **(C)** P2X_3_ and Polyphyllin V docking mode and the interaction plane diagram. **(D)** P2X_3_ and Polyphyllin F docking mode and the interaction plane diagram. **(E)** P2X_3_ and Polyphyllin VI docking mode and the interaction plane diagram. **(F)** P2X_3_ and Dioscin docking mode and the interaction plane diagram.

Molecular docking was applied to validate the binding of P2X_3_ receptors to seven active compounds shown in Table 3. Dioscin, Polyphyllin H, Polyphyllin I, Polyphyllin V, Polyphyllin VI, and Polyphyllin F are compatible with the structure of the P2X_3_ receptor, had binding energies of −12.0, −11.7, −11.6, −11.2, −10.8, and −10.6 kcal/mol, respectively, showing good binding of the receptor to the ligand by considering the absolute affinity value with >6 kcal/mol as the selected standard, implying a possible modulatory role (Figures 3A–F).

**TABLE 3:** Affinities and amino acid sites of ligand-protein detected by molecular docking.

Protein	Ligand	Binding energy kcal/mol	Amino acid binding site
P2X_3_	Dioscin	−12.0	ARG295/281, TYR285, TRP152, GLY129/130
	Polyphyllin H	−11.7	ARG295/529, GLN102, TRP152, GLU109
	Polyphyllin I	−11.6	TYR285, ARG295, GLN102, TRP152
	Polyphyllin V	−11.2	GLU109/156, GLY129/130, ARG295, TYR285
	Polyphyllin VI	−10.8	ARG295/281, TYR285, TRP152, GLY129/130
	Polyphyllin F	−10.6	ARG295/281, TYR285, TRP152, GLU109
	Gracillin	−5.7	ARG25, GLY314, ASP248

### 3.3 Effects of the PPVI on thermal withdrawal latency and mechanical withdrawal threshold in CFA-induced pain mice

**FIGURE 4:**
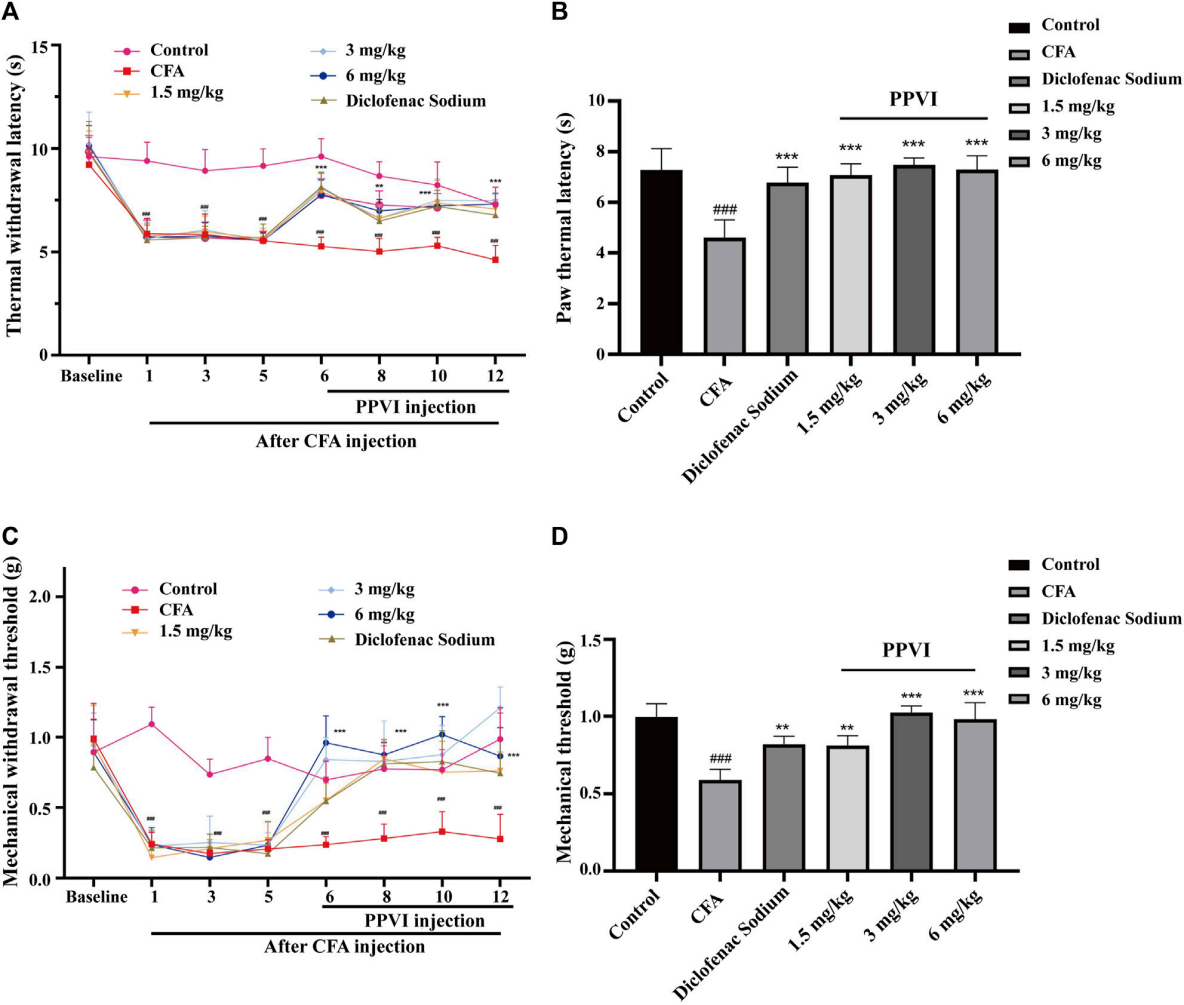
Effect of administration of PPVI on the TWL and MWT in CFA-induced chronic neuroinflammatory pain mice. **(A)** The TWL of mice in each group were detected on the day before CFA injection, 1, 3, 5 days after injection, and 1, 3, 5, and 7 days after PPVI admistration, *n* = 10 in each group. **(B)** THe TWL of mice in ache group were detected 12 days after CFA injection. **(C)** The MWT of mice in each group were detected on the day before CFA injection, 1, 3, 5 days after injection, and 1, 3, 5, and 7 days after PPVI administration. *n* = 10 in each group. **(D)** The MWT of mice in each group was detected 12 days after CFA injection. Data are presented as mean ± S.E.M, significant differences among different groups are indicated as ###*p* < 0.001, vs. control; **p < 0.001 vs. CFA group.

The results of TWL and MWT are shown in Figure 6 and the TWL (Figure 4A) and MWT (Figure 4C) were assessed in mice before modeling on days 1, 3, and 5 after the CFA injection. We found significant differences between the CFA group, the PPVI treatment group (1.5 mg/kg, 3 mg/kg, 6 mg/kg), and the diclofenac sodium group as compared to the control group, supporting the validity of the CFA paradigm. The TWL (Figure 4B) and MWT (Figure 4D) of mice in the PPVI administration group and the diclofenac sodium group were significantly higher than that of mice in the CFA group (*p* < 0.001). In the 1.5 mg/kg PPVI administration group analgesic effect was comparable to that of the diclofenac sodium group.

### 3.4 Effects of PPVI on foot swelling in CFA-induced chronic neuroinflammatory pain mice

**FIGURE 5:**
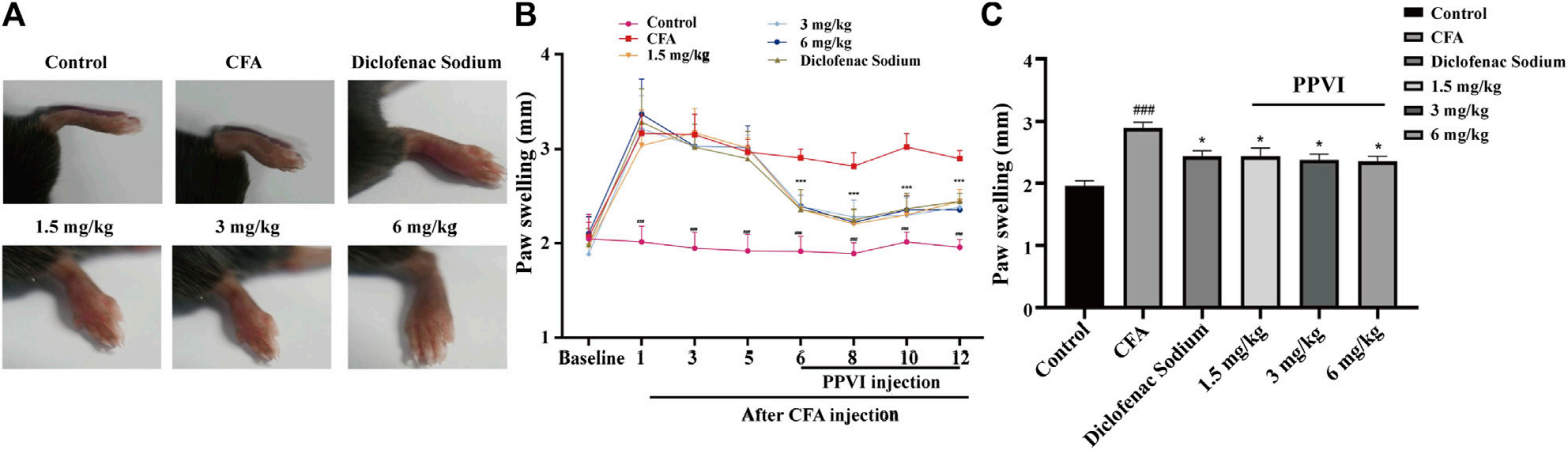
Effect of administration of PPVI on the foot swelling of CFA-induced chronic neuroinflammatory pain mice. **(A)** The foot swelling of mice in each group was detected on the 12 days after CFA injection. **(B)** The foot swelling of mice in each group was detected on the day before CFA injection, 1, 3, and 5 days after injection, and 1, 3, 5, and 7 days after PPVI administration, *n* = 10 in each group. **(C)** The foot swelling of mice in each group was detected 12 days after the CFA injection. Data are presented as mean ± S.E.M, significant differences among different groups are indicated as ###*p* < 0.001, vs. control; *p < 0.05 vs. CFA group.

The paw swelling was assessed in mice before pharmacological treatment on days 1, 3, and 5 following the CFA injection. The paws of the mice were swollen with significant differences (Figure 5) between the CFA, PPVI treatment group (1.5 mg/kg, 3 mg/kg, and 6 mg/kg), and diclofenac sodium group as compared to the control group, proving the validity of the CFA paradigm. Then, for 7 days, the PPVI group and the diclofenac sodium group received PPVI and diclofenac sodium injections, while the CFA and control groups received normal saline injections, while the left paw swelling was continuously monitored. The paw swelling of the mice in the PPVI groups (1.5 mg/kg, 3 mg/kg, and 6 mg/kg) and the diclofenac sodium group was significantly attenuated (Figures 5A–C).

### 3.5 Effects of PPVI on the expression of pro-inflammatory factors in CFA-induced chronic neuroinflammatory pain mice

**FIGURE 6:**
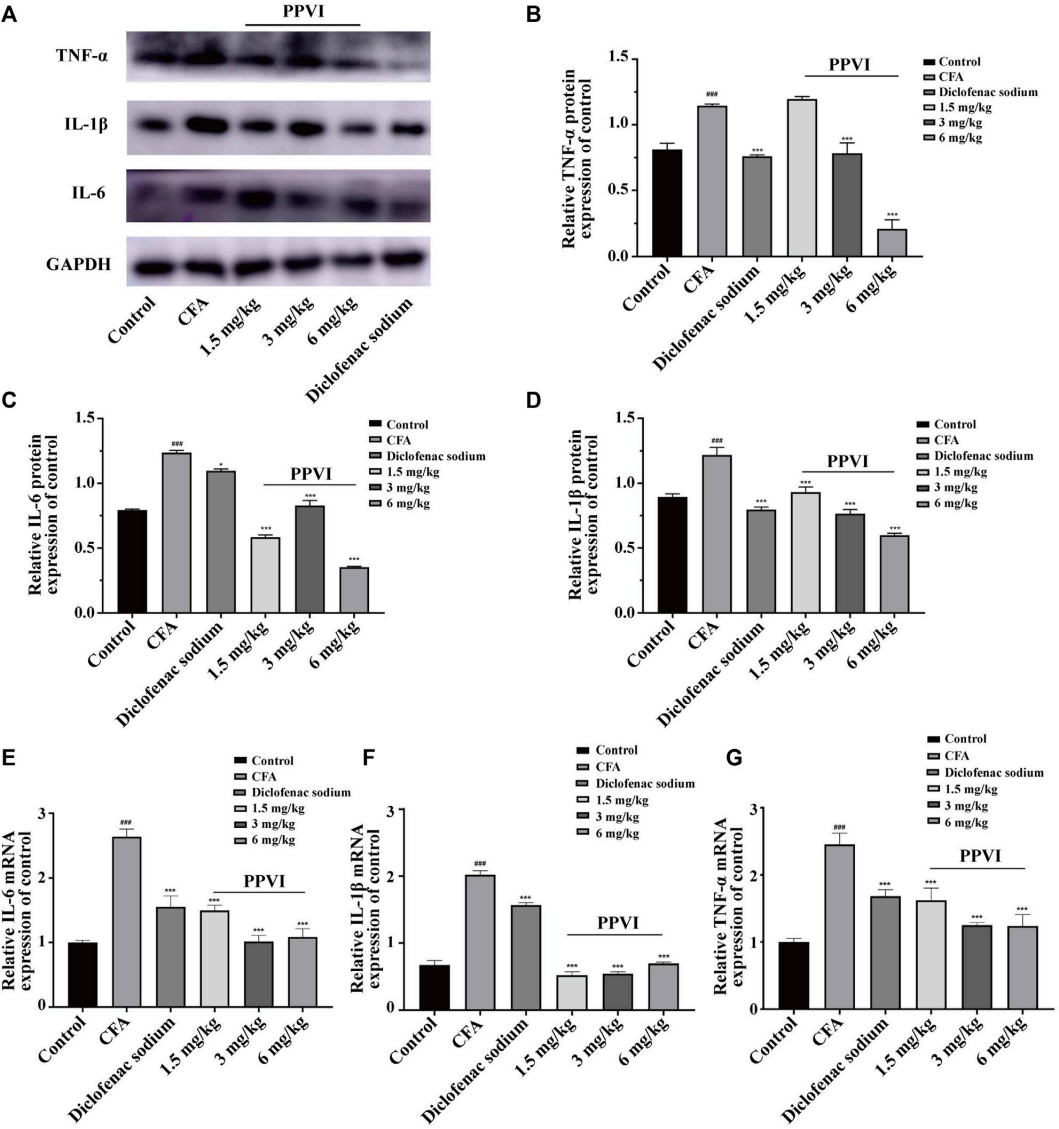
Effect of administration of PPVI on the expression of inflammatory factors IL—1β, IL-6, TNF-α in CFA-induced chronic neuroinflammatory pain mice. **(A–D)** The protein expression of inflammatory factors IL—1β, IL-6, and TNF-α in each group (*n* = 10). **(E–G)** The mRNA expression of inflammatory factors IL—1β, IL-6, and TNF-α in each group (*n* = 10). Data are presented as mean ± S.E.M, significant differences among different groups are indicated as ###*p* < 0.001, vs. control; *p < 0.05, **p < 0.01, ***p < 0.001 vs. CFA group.

To verify the anti-inflammatory effect of PPVI on DRGs and the spinal cord in CFA-induced mice, we performed assays using WB and qPCR. The results showed that the protein expression (Figures 6A–D) and mRNA expression (Figures 6E–G) of IL-1β, IL-6, and TNF-α of DRGs and spinal cord in CFA-induced mice were significantly increased compared with the control group. However, after 7 days of treatment with PPVI (1.5 mg/kg, 3 mg/kg, and 6 mg/kg), the expression of the IL-1β, and IL-6, TNF-α were normalized compared with the CFA-induced mice group.

### 3.6 Effects of PPVI on neural cells in DRG and spinal cord in CFA-induced inflammatory pain mice

**FIGURE 7:**
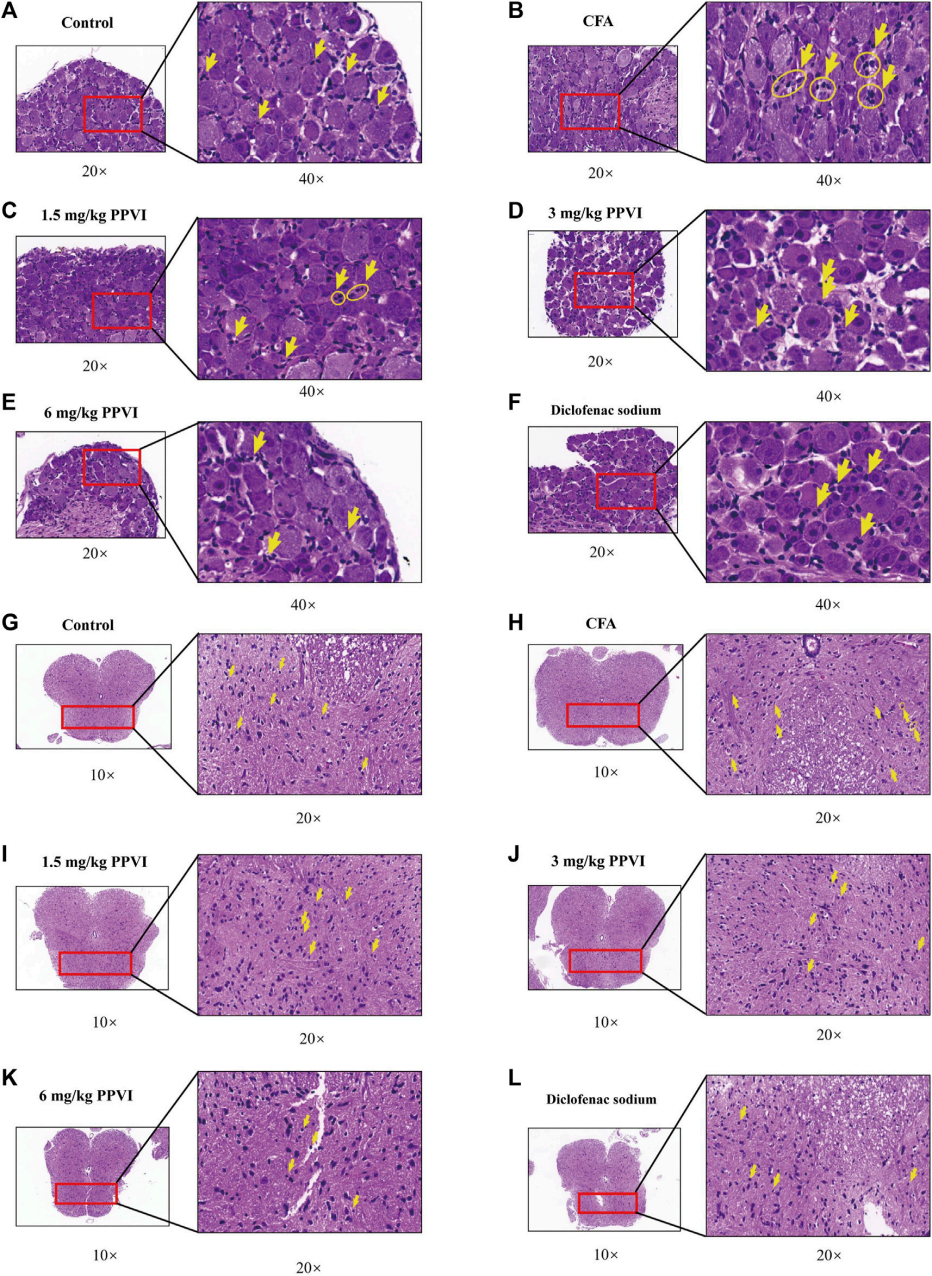
HE staining of DRG and spinal cord. **(A–F)** The HE staining of DRG in nerve cells in each group was detected by microscope (×200, ×400). **(G–L)** The HE staining of the spinal cord in nerve cells in each group was detected by microscope (×100, ×200).

HE-stained DRG and spinal cord sections are shown in Figure 7. Few inflammatory cells were observed in L_3_-L_5_ DRGs, while in the CFA group DRGs contained neutrophils, lymphocytes and macrophages gathered into small clusters (Figures 7A, B). After treatment with 1.5 mg/kg, 3 mg/kg, and 6 mg/kg PPVI, the presence of these inflammatory cells was significantly reduced in L_3_-L_5_ DRGs (Figures 7C–F). The distribution of glial cells is relatively uniform in the dorsal horn of the L_3_-L_5_ spinal cord of the control group. In contrast in the CFA group, aggregation of glial cells with pyknosis and vacuolization of neurons were observed in the dorsal horn of the L_3_-L_5_ spinal cord (Figures 7G, H indicated with an arrow). However, treatment with 1.5 mg/kg, 3 mg/kg, and 6 mg/kg PPVI reversed these changes (Figure 7I–L).

### 3.7 PPVI affects the expression of P2X_3_ receptors in DRG of CFA pain mice

**FIGURE 8:**
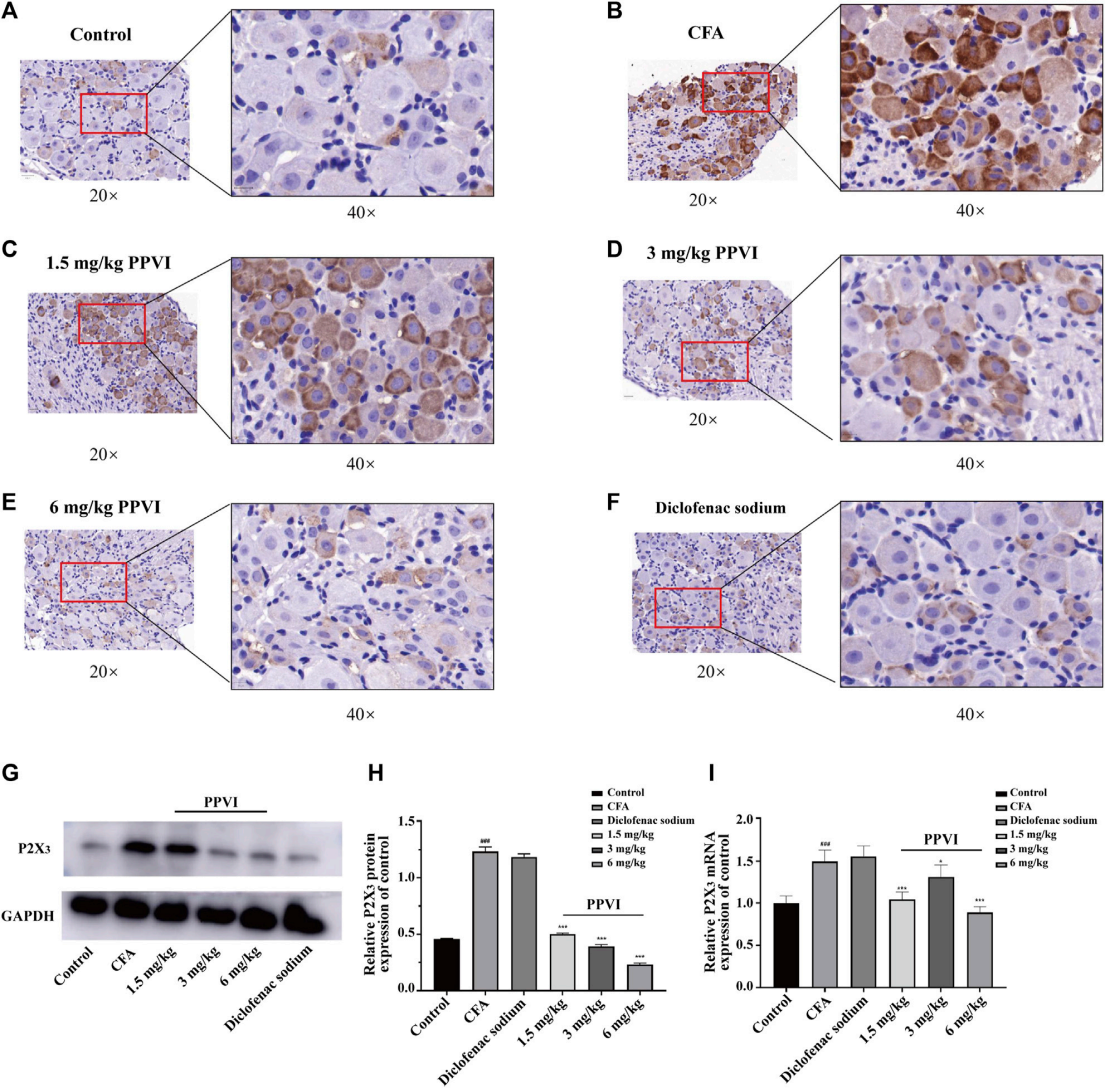
Effect of administration of PPVI on the expression of P2X_3_ of DRG in CFA-induced chronic neuroinflammatory pain mice. **(A–F)** The expression of P2X_3_ in nerve cells in each group was detected by immunohistochemistry (*n* = 3). **(G–I)** The protein and mRNA expression of P2X_3_ detected by western blot and qPCR in each group (*n* = 10). Data are presented as mean ± S.E.M, significant differences among different groups are indicated as ###*p* < 0.001, vs. control; *p < 0.05, **p < 0.001 vs. CFA group.

Immunohistochemistry showed that the expression of P2X_3_ receptors in L_3_-L_5_ DRGs from CFA mice was significantly increased compared with the control group (Figures 8A, B). However, treatment with PPVI (1.5 mg/kg, 3 mg/kg, and 6 mg/kg) and diclofenac sodium restored the expression of P2X_3_ receptors to control levels (Figures 8C–F) which is a significant difference compared with the CFA group. Further WB analysis showed that expression of P2X_3_ in DRGs was significantly upregulated in CFA mice compared with the control group and treatment with PPVI (1.5 mg/kg, 3 mg/kg, and 6 mg/kg) and diclofenac sodium restored the expression of P2X_3_ receptors in L_3_-L_5_ DRGs (Figures 8G–I).

### 3.8 PPVI normalized expression of P2X_3_ receptors in the dorsal horn of the spinal cord in CFA-induced pain mice

**FIGURE 9:**
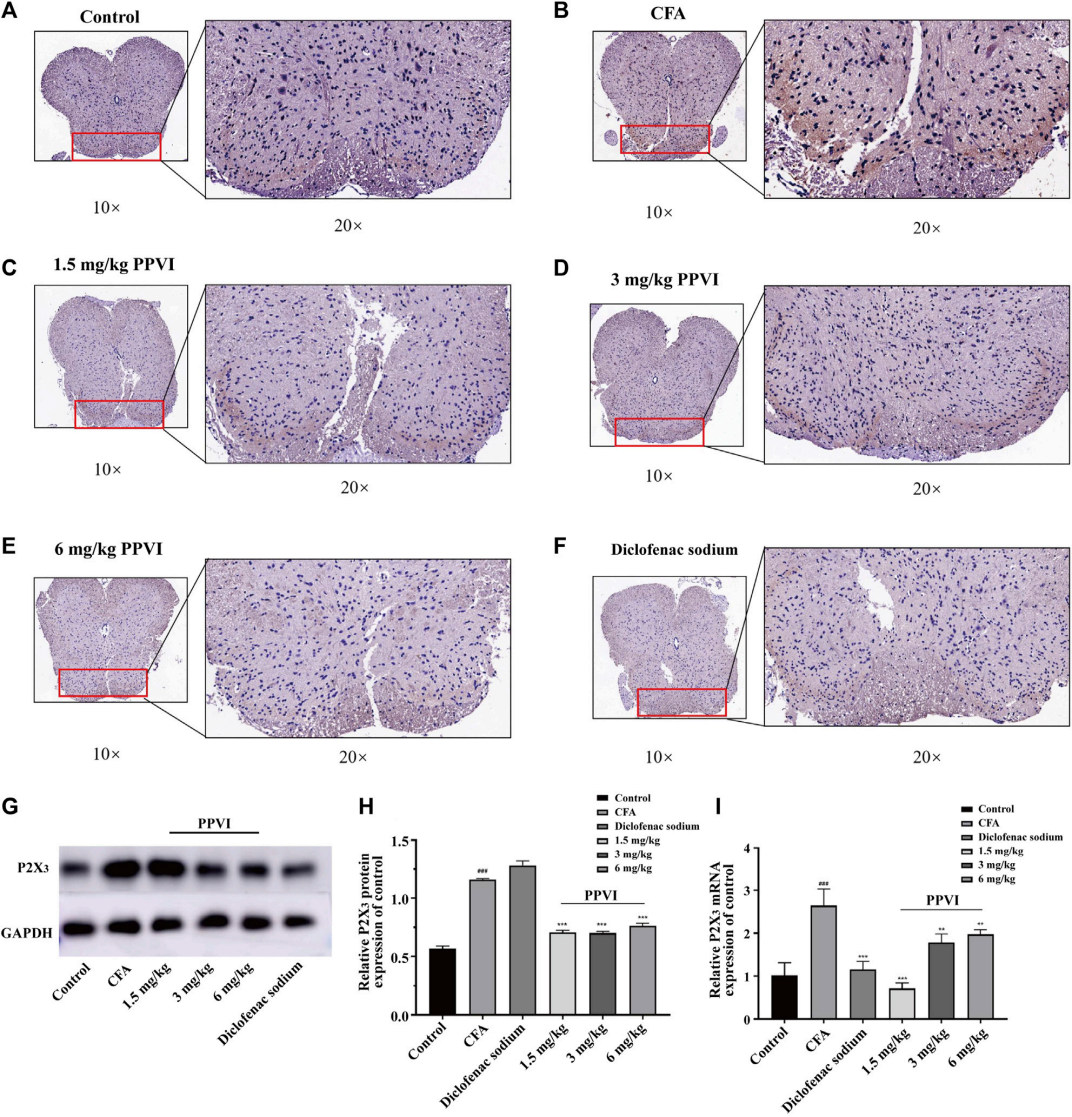
Effect of administration of PPVI on the expression of P2X_3_ of the spinal cord in CFA-induced chronic neuroinflammatory pain mice. **(A–F)** The expression of P2X_3_ in nerve cells in each group was detected by immunohistochemistry (*n* = 3). **(G–I)** The protein and mRNA expression of P2X_3_ detected by western blot and qPCR in each group (*n* = 10). Data are presented as mean ± S.E.M, significant differences among different groups are indicated as ###*p* < 0.001, vs. control; *p < 0.05, **p < 0.01, ***p < 0.001 vs. CFA group.

We also analyzed the effect of PPVI on the expression of P2X_3_ receptors in the L_3_-L_5_ spinal cord of CFA-induced pain mice. Compared with the control group, the expression of P2X_3_ receptors in the dorsal horn of the spinal cord of CFA mice was significantly upregulated (Figures 9A,B). However, treatment with PPVI (1.5 mg/kg, 3 mg/kg, and 6 mg/kg) and diclofenac sodium restored the expression of P2X_3_ receptors (Figures 9C–F). Further WB analysis showed increased protein expression of P2X_3_ receptors in the dorsal horn of CFA mice compared with the control group. However, treatment with PPVI (1.5 mg/kg, 3 mg/kg, and 6 mg/kg) and diclofenac sodium restored the protein expression of P2X_3_ receptors (Figures 9G–I).

## 4 Discussion

*Polyphylla* var. *yunnanensis* is widely used as an anti-tumor treatment in traditional Chinese medicine (He et al., 2015). A recent study found that Rhizoma Paridis saponins extract from *Polyphylla* var. *yunnanensis* demonstrates analgesic effects in a mouse model of chronic cancer pain (Wang G. et al., 2018), although underlying mechanisms and the active ingredients of *Polyphylla* var. *yunnanensis* remain unknown. In this study, the *Polyphylla* var. *yunnanensis* extract contained seven main active ingredients: Dioscin, Polyphyllin H, Polyphyllin I, Polyphyllin V, Polyphyllin VI, and Polyphyllin F. In glioblastoma or glial cells, little or no P2X_3_ receptors were expressed since P2X_3_ is specific for sensory neurons (Inoue and Tsuda, 2021). Therefore, we used the U373 cells overexpressing P2X_3_ receptors to compare the differences between the U373 cells overexpressing P2X_3_ and normal U373 cells to observe the dissociated fluid components caused by the increase of P2X_3_ receptors. Combining the results of the docking of P2X_3_ and active molecules, we initially screened the main active ingredient polyphyllin VI in the Chonglou extract that may have a regulatory effect on the P2X_3_ receptor.

A suspension of whole or crushed, heat-inactivated mycobacteria is present in mineral oil that is used to make CFA (Stills, 2005). Stimulation of the immune response, which results in delayed hypersensitivity at the injection site, as well as significant inflammatory reactions and hyperalgesia induces chronic pain. (Sadler et al., 2022).

Changes in functional expression of P2X_3_ receptors are closely related to inflammation, while CFA injections or chronic nerve compression and temporal mandibular joint disorders increase the expression of P2X_3_ receptors (Xu and Huang, 2002; Ambalavanar et al., 2005; Shinoda et al., 2005). Intrathecal injection of P2X_3_ receptor agonist α, *β*-meATP enhances pain behavior (Xiang et al., 2008), while intrathecal injection of P2X_3_ receptor antagonist A-317491 or treatment with antisense P2X_3_ receptor oligonucleotides significantly reduces formalin- or α,*β*-meATP injection-induced nociceptive behavior and nociceptive inflammatory response in DRG and spinal cord after partial sciatic nerve ligation, CFA adjuvant, formalin or α,*β*-meATP injection into the skin (Barclay et al., 2002; Hemmings-Mieszczak et al., 2003; McGaraughty et al., 2003; Pissanetzky, 2016). In addition, inflammatory cytokines IL-1, IL-6, and TNF-α also contribute to the pathophysiology of chronic pain (del Rey et al., 2012; Li et al., 2017; Yang et al., 2020). Inflammatory cytokines have a role in peripheral inflammation, a key contributor to chronic pain. IL-1, IL-6, and TNF-α, that can trigger immunopathological reactions and intensify inflammatory signals, are particularly important (del Rey et al., 2012; Burnstock, 2016; Yang et al., 2020).

Several cell types in DRG and the spinal cord, including immune cells, neurons, and glial cells, produce IL-1 in response to peripheral nerve injury (Liddelow et al., 2017; Voet et al., 2019; Trapero and Martin-Satue, 2020). Increased expression of P2X_3_ receptors may potentiate the production of IL-1, which intensifies the inflammatory response of microglia in the spinal cord (Shieh et al., 2006). Hypomethylation of the P2X_3_ receptor gene promoter regions in rat tumor cells improves the binding of members of the NF-κB family of transcriptional regulators and increases pain sensitivity, which (Zhou et al., 2015). These studies suggest that inflammation can upregulate the expression of P2X_3_ in sensory nerves, while the activation of P2X_3_ can promote the secretion of inflammatory factors. This study found that PPVI reduced CFA mice paw edema and pain threshold, indicating a significant analgesic effect. Further experiments showed that PPVI reduced the number of inflammatory cells in DRG and downregulated the inflammatory factors TNF-α, IL-1β, and IL-6 expression in DRG and spinal cord of CFA mice. Subsequently, we demonstrated that PPVI inhibited P2X_3_ expression in the DRG and spinal cord of CFA mice, indicating that the analgesic effect is linked to the decrease in P2X_3_ receptors expression and the decrease in inflammatory cytokines. However, further research is still needed to investigate how PPVI regulates the signaling pathway between P2X_3_ and inflammation in DRG and the spinal cord.

## 5 Conclusion

In summary, by using molecular docking technology and U373 cells overexpressing P2X_3_ receptors combined with the cell membrane immobilized chromatography we identified PPVI as the main active component of CL. PPVI increases the mechanical and thermal withdrawal threshold of CFA-induced pain mice and relieves the pain and foot swelling. In addition, PPVI downregulates the expression of TNF-α, IL-1β, and IL-6 in DRG and spinal cord to alleviate the inflammation; PPVI also normalizes the expression of P2X_3_ purinoceptors. Our work provides insight into the potential new targets of PPVI for the treatment of inflammatory pain.

## Data Availability

The datasets presented in this study can be found in online repositories. The names of the repository/repositories and accession number(s) can be found in the article/supplementary material.
